# Pacific Islands Families Study: Household Food Security during Pregnancy and Secondary School Educational Achievement

**DOI:** 10.3390/nu15194131

**Published:** 2023-09-25

**Authors:** Leon Iusitini, El-Shadan Tautolo, Lindsay D. Plank, Elaine Rush

**Affiliations:** 1 New Zealand Work Research Institute, Faculty of Business, Economics and Law, Auckland University of Technology, Auckland 1142, New Zealand; leon.iusitini@aut.ac.nz; 2 School of Public Health & Interdisciplinary Studies, Faculty of Health and Environmental Sciences, Auckland University of Technology, Auckland 1142, New Zealand; dan.tautolo@aut.ac.nz; 3 Department of Surgery, Faculty of Medical and Health Sciences, University of Auckland, Auckland 1142, New Zealand; l.plank@auckland.ac.nz; 4 School of Sport and Recreation, Faculty of Health and Environmental Sciences, Auckland University of Technology, Auckland 1142, New Zealand

**Keywords:** food insecurity, birth cohort, academic achievement, Pacific Islands

## Abstract

Nutritional environment in early life is a key factor for brain development and function. It is important to understand the relationship between nutrition in early life and academic achievement in adolescence. The birth cohort of the Pacific Islands Families (PIF) study was born in the year 2000. When their child was six weeks old, mothers were asked questions concerning food security over the past year. Two binary measures of food security were derived as previously used in PIF and also by the Ministry of Health (MOH). In 2020, records of academic achievement from the National Certificate of Educational Achievement (NCEA) for 649 (317 female, 332 male) cohort members showed progressive achievement at levels 1, 2, and 3 of NCEA and allowed University Entrance (UE) to be assessed. The prevalence of food insecurity was not different for sex but high at 29% and 42% using the PIF and MOH definitions of food insecurity, respectively. More females (27%) than males (18%) achieved UE as their highest qualification, and more males (40%) than females (31%) achieved NCEA levels 1 or 2 as their highest qualification. UE was achieved by 25% of those born into food-secure households and 17% from food-insecure households. Logistic regression demonstrated that the odds of achieving UE were 1.8-fold (95% CI 1.2, 2.6, *p* = 0.003) higher in females than males and, independently, 1.6-fold (95% CI 1.1, 2.5 *p* = 0.026) higher if the household was food secure. This work emphasises the importance of maternal and early-life food security for subsequent academic achievement and the well-being of future generations.

## 1. Introduction

Maternal nutrition is fundamental to child growth and development during gestation [1,2]. Nutrient supply from the time of conception to the age of two years (i.e., the first 1000 days after conception) lays the foundation for the child’s future health and wellbeing [3], with diet composition impacting fetoplacental growth and metabolic patterns [4]. The development of the brain and cognition for future academic achievement is dependent on nutritional factors and stimulation from the environment [5]. Food insecurity increases the risk of malnutrition as it is associated with the consumption of less healthy combinations of foods, and the over- and under-consumption of macro- and micro-nutrients are essential for health. At an individual level, food insecurity in New Zealand is associated not only with low income, ethnic considerations, and socioeconomic deprivation [6] but also with less variety in the diet [7]. Households experiencing food insecurity are more likely to purchase poorer quality or fewer foods from the vegetable, fruit, and cereal groups [8]. In turn, a better-quality maternal diet during pregnancy has a small positive association with neurodevelopment in the child [9].

Throughout life, the molecules that make up the structure and determine the function of the human body are derived from the environment: specifically, food, water, and air. In addition, when cells are rapidly dividing and differentiating, the way that genes are expressed can be altered by exogenous factors that affect function, including metabolic pathways and the development of the brain and neurocognitive pathways [10]. A causal mechanism requires a biological pathway and process that can bring about an outcome. These pathways originate early in life, and the first 1000 days are a critical period [11] when the availability of nutrients makes a critical difference in growth. Determinants of household food insecurity, such as marital status, age, sex, ethnicity, and household income, confound one another [12] in the prediction of food insecurity. Multivariate analysis shows that experiencing a low income is the strongest predictor of household food insecurity in New Zealand [12]. These “upstream” effects, or socioeconomic determinants of health, are part of the pathway, but the proximal biological explanation, or causality at the physical growth and developmental level, is the quality and quantity of nutrient supply [4].

It has been shown previously that educational achievement Is associated with food insecurity in longitudinal and cross-sectional studies [13,14]. No studies, however, appear to have directly assessed the relationship between educational outcomes in youth and maternal food insecurity in the period encompassing conception and gestation.

The longitudinal Pacific Islands Families (PIF) study commenced in the year 2000 when the mothers of 1398 Pacific infants born at Middlemore Hospital in South Auckland consented to take part [15]. Since that time, this study has tracked the health and development of the cohort with follow-up at ages 6 weeks, 1, 2, 4, 6, 9, 11, 14, 18, and 22 years. At six weeks post-partum, almost half (43.5%) of the 1376 mothers reported that in the preceding year, they either sometimes or often “ran out of food due to lack of money” [7]. In a nested sub-study, at the age of 14 years, we found that for boys but not girls, food insecurity during gestation was associated with less skeletal muscle mass and increased fat mass (measured by whole-body DXA) [16]. Further analysis at age 14 years for 931 cohort members (463 female) [17] showed that females were 1.9-fold more likely to be obese according to body mass index criteria if they were categorised as food insecure during gestation. 

In 2020, the New Zealand Qualifications Authority (NZQA) provided the PIF Study research team with individual measures of secondary school educational achievement for cohort members who gave consent. The objective of this analysis was to determine and quantify the existence of relationships between household food security during gestation and academic achievement between the secondary school ages of 15 and 19 years.

## 2. Materials and Methods

Data from the Pacific Islands Families study were initially collected in the year 2000 at 6-weeks post-partum with ethics approval from the National Ethics Committee, Auckland branch (ref. 99/055). Mothers provided informed written consent and answered questions concerning food security over the previous year as part of a broader interview on their child’s health, development, and family circumstances. Between 2016 and 2019, as part of wider data collection, cohort members provided written consent for their educational achievement records to be obtained from the NZQA (Health and Disability Ethics Committee approval numbers 17/CEN/262).

### 2.1. Household Food Security

At the 6-week post-partum maternal interview, responses to seven questions about household food security over the preceding year (Table 1) were answered as either never, sometimes, or often and were scored 0, 1, and 2, respectively. These scores were added up, and those with a score of ≥4 out of a possible 14 were categorised as food insecure [16,18]. In addition, in the National Health Survey, [6] the Ministry of Health (MOH) scored food security based on only the first of these seven questions: “Food runs out in our household due to lack of money. How often has this been true for your household over the past year?” Responses were “often, sometimes or never”. If answered “often”, or “sometimes”, the household was classified as food insecure, yielding a MOH food security binary variable.

### 2.2. Academic Achievement

In New Zealand, the main secondary school qualification is the National Certificate in Educational Achievement (NCEA). NCEA is based on assessment standards of either unit standards or achievement standards at levels 1 to 3 for secondary school. Unit standards are competency-based (achieved or not achieved) and apply to vocational settings. For achievement standards, the following grades are awarded: not achieved, achieved, merit, or excellence. Generally, students in year 11 undertake level 1, year 12 undertake level 2, and year 13 undertake level 3. Standards have defined credit values where one credit value represents approximately 10 h of teaching, learning, and assessment. A total of 80 credits at each level are required to be awarded a certificate at that level [19,20]. University Entrance (UE) is an additional award based on the achievement of certain level 3 standards approved by NZQA. Raw data were received from NZQA on individual qualifications attained, educational standards attempted, and results achieved, as well as any endorsements/grades recognising high achievement at the certificate level and in individual courses/subjects for the academic years 2014 through 2019. 

An overall NCEA achievement score variable was derived from information about the achievement standards attempted and achieved by the cohort. The standards from levels 1, 2, and 3 with a result of achieved, merit, or excellence were used to calculate the overall score. For each participant, the 60 best credits from the dataset mentioned above for level 1, the 60 best credits for level 2, and the 60 best credits for level 3 (ranked in order of excellence, merit, and achievement) were identified. For each of these three levels, a score using the 60 best credits was calculated using 4 × [excellence credits] + 3 × [merit credits] + 2 × [achieved credits]. The overall NCEA achievement score was calculated by adding the scores from level 1, level 2, and level 3 together. This allowed for a minimum score of 0 and a maximum score of 720 (the highest score a participant received was 719). Within each level, a minimum score of 0 and a maximum score of 240 could be achieved.

### 2.3. Statistics

Continuous variables are not normally distributed, so Mann–Whitney U-tests were used to compare NCEA achievement with the child’s sex and food security as independent variables. The median and interquartile range (IQR) are reported. Chi-squared tests were used for categorical data. Logistic regression was used to compute unadjusted and adjusted odds ratios. Statistical analyses were conducted with IBM SPSS Statistics Version 29.0.0.0. Reported probabilities were two-sided, and *p*-values < 0.05 were considered statistically significant.

## 3. Results

In total, 650 cohort members consented to participate, and their NCEA data were accessed. One participant lacked sufficient NCEA data to ascertain their educational achievement, so the analysis included 649 youths (317 females, 332 males). There were no significant differences in the responses to the food security questions between the full cohort and this analysis (Table 1). In this analysis, at birth, 32% of households with female offspring and 27% of households with male offspring were rated food insecure (*p* = 0.168) using previously published PIF criteria [16], while 43% of households with female offspring and 40% of households with male offspring were categorised as food insecure using MOH criteria (*p* = 0.438) (Table 1).

Of the 649 cohort members, 65 (10%, 28 female 37 male) had no qualifications recorded in the NZQA qualifications dataset, and proportions with no qualifications did not differ significantly by sex (*p* = 0.329). More females (27%) than males (18%) achieved University Entrance as their highest qualification, and more males (40%) than females (31%) achieved NCEA levels 1 or 2 as their highest qualification (Table 2).

The proportion of youths who achieved NCEA levels 1 or 2 as their highest qualification was higher for those categorised as food insecure. A higher proportion of food-secure youths (25%) achieved University Entrance than those categorised as food insecure (17%) (Table 3).

For both females and males, achievement points overall and at each level were higher if they came from a food-secure household during gestation (Table 4 and Table 5). However, statistically significant differences at each level of achievement were only seen in females, not males. More points were gained at level 1 than at level 3 for each sex.

Logistics regression was applied to assess the influence of sex and household food security on the achievement of a UE qualification. The odds of achieving UE were 1.8-fold (95% CI 1.2, 2.6, *p* = 0.003) higher in females than males and, independently, 1.6-fold (95% CI 1.1, 2.5 *p* = 0.026) higher if the household was food secure according to the PIF study definition (Table 6). This finding was very similar when the MOH definition for food security was applied (Table 6).

## 4. Discussion

This analysis has shown that food security in the year preceding birth and female sex are independently associated with higher academic achievement at secondary school. These findings are important as the prevalence of food insecurity in households with children in New Zealand over the past two decades has been persistently high. In the 2002 National Children’s Nutrition Survey, 18.1% of New Zealand households with children reported that they often or sometimes ate less due to lack of money, but this proportion was 48% among households with Pacific children [21]. In the 2012 and 2021/2022 National Health Surveys, 24% and 13% of respondents reported eating less due to a lack of money often or sometimes, respectively, but for Pacific households, these respective proportions were 50% and 38% [6]. While there was some reduction in the prevalence of food insecurity in the decade preceding 2021/22, in the year leading up to June 2023, food prices increased by 12.5%, with vegetable and fruit prices increasing by 22% [22]; therefore, it is unlikely that this improvement will continue. This analysis has shown that food insecurity during gestation precedes and is statistically linked to poorer educational outcomes later in life and contributes evidence to the need for a life course approach to reduce disparities [23] and alter the life course trajectory.

This study demonstrates the effect of previous exposure (food security) on later outcomes (educational attainment). A New Zealand report [24] that looked at the contribution schools make to NCEA achievement also looked at the influence of parental education, sex, and home environment, as well as socioeconomic factors, on achievement within schools. The report concluded that parents’ educational attainment is the strongest predictor of students’ NCEA achievement in New Zealand, and schools, for the most part, are not the cause of differences in student’s achievement. In other words, parental educational attainment, particularly the mothers’, is linked to the educational outcomes of their offspring. Parental education, in turn, is associated with family income, which is the proximal driver of food security.

Food insecurity is a relentless problem as causation is complex, with multiple and diverse drivers [25], while some interventions can also aggravate problems in the food system. For example, the zero-rating of goods and services tax on food might not benefit those most in need of food and allow profit margins to be increased. However, the proximal and immediate solution is to enable more income to buy food while, at the same time, improving the equity of geographical access to food.

This analysis shows that 27% of females and 18% of males, or 23% of the PIF study cohort overall, achieved University Entrance, which is slightly less than the national rate of University Entrance attainment by Pacific students in 2017 (29%) [26]. The statistics for the year 2017 are quoted because this is the year when the PIF cohort was 17 years old: the usual age of achievement of University Entrance by year 13 students. The finding that males had lower educational achievement than females at the same age is also recorded in national statistics where, for example, in 2017, 31% of males and 47% of females achieved University Entrance [26]. This sex difference could be related to the increased sensitivity of the male placenta to adverse nutritional exposures [27] and the fact that, from early gestation, males grow more rapidly than females [28] and are more sensitive to environmental stressors. In the PIF study cohort, at the age of 14 years, sexual dimorphism was demonstrated in that males exposed to gestational food insecurity were more likely to have more fat, more visceral adipose tissue, and less appendicular skeletal muscle mass than females [16].

### 4.1. Limitations and Strengths

This analysis is limited to household food insecurity during the year before birth, which includes gestation and secondary school educational achievement 15 or more years later. It is likely that food insecurity continued within these families through the life course of the child, exacerbated by the birth of more siblings and the fact that as children grow, they cost more to feed. The measures of household food security were subjective and based on measures in national health surveys to enable comparisons to be undertaken. The questions used to assess household food security have previously demonstrated construct validity with nutritional status and ability to rank households according to the severity of food insecurity [18]. No further measures of food insecurity were undertaken in the PIF study cohort, but dietary quality in this cohort was examined at ages 4 and 6 years [29]. The consumption of food increased from 4 to 6 years, but the frequency of consumption for vegetables, fruit, and milk decreased, while cereals, rice, and bread (mainly white and refined) were stable. The fall in vegetable and fruit consumption with age is also observed in the National Health Survey [6], which could reflect the increasing cost and amount of these foods required for the family. Low vegetable and fruit intake is associated with low incomes and higher deprivation [30].

A strength of this study is that this is a birth cohort study design with measures of food security for the year prior to birth. The participants were of Pacific ethnicity and represented a population with a high prevalence of food insecurity in relation to having “not enough” money for food. The use of NCEA educational data is also a strength, as academic performance was objectively tracked over 3 or more years. On the other hand, some students could take a more vocational pathway and not take units that lead to UE; therefore, the attainment of UE is not necessarily a reflection of academic ability. 

### 4.2. Future Work

The participants in this study are now 23 years old, and some are parents already. It is important that this intergenerational cycle of inadequate nutrition, food insecurity, and inequitable academic achievement is broken and that future children have a better start to life. This requires a holistic and equity-focused approach that looks at the interrelated factors that affect deprivation, including food production, distribution, supply, and cost at personal, familial, societal, and political levels. This work emphasises the importance of maternal food security for the well-being of future generations, as higher levels of education are associated with higher income and better health status. It also points to the need to factor in sex differences when considering interventions. At global and national levels, the realisation of sustainable development goals is necessary to achieve a vision of food security for all [31].

## Figures and Tables

**Table 1:** Maternal responses to food security questions at 6 weeks post-partum for both the full cohort and this analysis.

	This Study (*n* = 649)			Full Cohort (*n* = 1398)		
	**Female** **(*n* = 317)**	**Male** **(*n* = 332)**	***p***	**Female** **(*n* = 681)**	**Male** **(*n* = 717)**	***p***
Food runs out due to lack of money	57/40/3	60/38/3	0.749	55/40/5	58/39/3	0.070
2. I/we eat less because of lack of money	62/34/4	67/30/3	0.368	61/34/5	66/32/2	0.026
3. The variety of foods I am (we are) able to eat is limited by lack of money	58/36/6	65/32/3	0.119	58/35/7	63/34/3	0.002
4. I/we rely on others to provide food and/or money for food, for my/our household when I/we don’t have enough money	67/32/2	71/27/2	0.367	68/29/3	72/26/2	0.332
5. I/we make use of special food grants or food banks when I/we do not have enough money for food	85/15/0	89/11/0	0.257	85/14/1	87/13/0	0.223
6. I feel stressed because of not having enough money for food	63/31/6	69/28/3	0.175	63/32/6	68/29/3	0.052
7. I feel stressed because I can’t provide the food I want for social occasions	73/24/4	78/20/2	0.131	73/23/4	78/20/2	0.025
	% food secure/food insecure					
Total for 7 questions ≤3/≥4	68/32	73/27	0.168	65/35	71/29	0.027

To each question, mothers responded “never”, “sometimes”, or “often”, which were scored 0, 1 or 2, respectively. A total score was calculated by adding all seven individual question scores. Total score ≤ 3 was categorised as food secure and ≥4 as food insecure.

**Table 2:** Highest secondary school qualification according to sex.

	Overall *n* = 649	Female *n* = 317	Male *N* = 332	*p* *
**No qualification recorded**	65 (10.0)	28 (8.8)	37 (11.1)	0.329
**NCEA Level 1 or 2**	233 (35.9)	99 (31.2)	134 (40.4)	0.015
**NCEA Level 3**	204 (31.4)	103 (32.5)	101 (30.4)	0.565
**University Entrance**	147 (22.6)	87 (27.4)	60 (18.1)	0.005

Data are the number (%) * Chi-squared test for comparison of proportions.

**Table 3:** Highest secondary school qualification according to food security status (PIF Study definition) during pregnancy.

	Food Secure *n* = 459	Food Insecure *n* = 190	*p* *
No qualification recorded	47 (10.2)	18 (9.5)	0.787
NCEA Level 1 or 2	157 (24.2)	69 (36.3)	0.002
NCEA Level 3	135 (29.4)	65 (34.2)	0.229
University Entrance	114 (24.8)	33 (17.4)	0.041

Data are the number (%) * Chi-squared test for comparison of proportions.

**Table 4:** Secondary school achievements according to sex and food security status (PIF study definition) during pregnancy.

Variable	Female		* *p*	Male		* *p*
	Food Secure (*n* = 216)	Food Insecure (*n* = 101)		Food Secure (*n* = 243)	Food Insecure (*n* = 89)	
Overall NCEA achievement score	428 (351, 532)	406 (271, 466)	0.032	385 (292, 460)	375 (292, 423)	0.332
Total points level 1	158 (130, 195)	145 (124, 181)	0.021	141 (124, 169)	136 (120, 158)	0.253
Total points level 2	146 (124, 174)	137 (120, 158)	0.044	133 (120, 159)	124 (118, 152)	0.067
Total points level 3	133 (80, 165)	124 (60, 151)	0.035	120 (38, 146)	120 (45, 136)	0.492

Data are the median (IQR) * Mann–Whitney U-test. NCEA, National Certificate of Educational Achievement. Overall NCEA achievement score is total points at levels 1, 2 and 3.

**Table 5:** Secondary school achievements according to sex and food security status (MOH definition) during pregnancy.

Variable	Female		* *p*	Male		* *p*
	Food Secure (*n* = 180)	Food Insecure (*n* = 137)		Food Secure (*n* = 198)	Food Insecure (*n* = 134)	
Overall NCEA achievement score	429 (361, 533)	411 (289, 468)	0.032	388 (295, 464)	373 (287, 429)	0.097
Total points level 1	159 (125, 178)	150 (126, 178)	0.044	144 (126, 174)	136 (120, 158)	0.047
Total points level 2	148 (124, 179)	138 (120, 160)	0.011	134 (120, 160)	127 (120, 158)	0.061
Total points level 3	132 (81, 173)	126 (60, 153)	0.066	127 (120, 160)	120 (40, 135)	0.144

Data are the median (IQR) * Mann–Whitney U-test. MOH, Ministry of Health; NCEA, National Certificate of Educational Achievement. Overall NCEA achievement score is total points at levels 1, 2 and 3.

**Table 6:** Unadjusted and adjusted odds ratios for Pacific Islands Families (PIF) study members who achieved a University Entrance qualification with sex and food security as independent variables.

Exposure	Exposed Achieved UE %	Not Exposed Achieved UE %	Unadjusted OR (95% CI)	Adjusted OR (95% CI)	*p*
Household food security by PIF study definition					
Sex (Female)	27.4	18.1	1.71 (1.18, 2.48)	1.76 (1.21, 2.56)	0.003
Food secure	24.8	17.3	1.58 (1.03, 2.43)	1.65 (1.07, 2.55)	0.026
Household food security by MOH definition					
Sex (Female)	27.4	18.1	1.71 (1.18, 2.48)	1.75 (1.20, 2.54)	0.004
Food secure	26.2	17.6	1.66 (1.13, 2.44)	1.70 (1.15, 2.51)	0.008

## Data Availability

Data sharing/access can be sought from the co-directors of the Pacific Islands Families study: c/- research@aut.ac.nz. Applicants are required to submit a concept paper outlining their analysis plan and their use of data, as well as a provision for secure storage and access to data if not held on-site.

## References

[1] Fitzgerald E., Hor K., Drake A.J. (2020). Maternal influences on fetal brain development: The role of nutrition, infection and stress, and the potential for intergenerational consequences. Early Hum. Dev..

[2] Arora N.K., Swaminathan S., Mohapatra A., Gopalan H.S., Katoch V.M., Bhan M.K., Rasaily R., Shekhar C., Thavaraj V., Roy M. (2017). Research priorities in Maternal, Newborn, & Child Health & Nutrition for India: An Indian Council of Medical Research-INCLEN Initiative. Indian J. Med. Res..

[3] Darling J.C., Bamidis P.D., Burberry J., Rudolf M.C.J. (2020). The First Thousand Days: Early, integrated and evidence-based approaches to improving child health: Coming to a population near you?. Arch. Dis. Child..

[4] Morrison J.L., Regnault T.R. (2016). Nutrition in Pregnancy: Optimising Maternal Diet and Fetal Adaptations to Altered Nutrient Supply. Nutrients.

[5] Prado E.L., Dewey K.G. (2014). Nutrition and brain development in early life. Nutr. Rev..

[6] Ministry of Health National Health Survey. https://minhealthnz.shinyapps.io/nz-health-survey-2021-22-annual-data-explorer.

[7] Rush E., Puniani N., Snowling N., Paterson J. (2007). Food security, selection, and healthy eating in a Pacific Community in Auckland New Zealand. Asia Pac. J. Clin. Nutr..

[8] Smith C., Parnell W.R., Brown R.C., Gray A.R. (2013). Balancing the diet and the budget: Food purchasing practices of food-insecure families in New Zealand. Nutr. Diet..

[9] Borge T.C., Aase H., Brantsaeter A.L., Biele G. (2017). The importance of maternal diet quality during pregnancy on cognitive and behavioural outcomes in children: A systematic review and meta-analysis. BMJ Open.

[10] Polverino A., Sorrentino P., Pesoli M., Mandolesi L. (2021). Nutrition and cognition across the lifetime: An overview on epigenetic mechanisms. AIMS Neurosci..

[11] Agosti M., Tandoi F., Morlacchi L., Bossi A. (2017). Nutritional and metabolic programming during the first thousand days of life. Pediatr. Med. Chir..

[12] Carter K.N., Lanumata T., Kruse K., Gorton D. (2010). What are the determinants of food insecurity in New Zealand and does this differ for males and females?. Aust. N. Z. J. Public Health.

[13] Shankar P., Chung R., Frank D.A. (2017). Association of Food Insecurity with Children's Behavioral, Emotional, and Academic Outcomes: A Systematic Review. J. Dev. Behav. Pediatr..

[14] Camelo K., Elliott M. (2019). Food insecurity and academic achievement among college students at a public university in the United States. J. Coll. Stud. Dev..

[15] Paterson J., Percival T., Schluter P., Sundborn G., Abbott M., Carter S., Cowley-Malcolm E., Borrows J., Gao W., the PIF Study Group (2008). Cohort profile: The Pacific Islands Families (PIF) Study. Int. J. Epidemiol..

[16] Oyama S., Tautolo E.-S., Tukuitonga C., Rush E. (2021). Pacific Islands Families Study: Adverse impact of food insecurity on child body composition. N. Z. Med. J..

[17] Rush E., Tautolo E., Plank L. Pacific Islands Families study: Food insecurity in pregnancy presages in adolescence obesity in girls and metabolic risk in boys. Proceedings of the International Congress of Obesity.

[18] Parnell W.R., Gray A.R. (2014). Development of a food security measurement tool for New Zealand households. Br. J. Nutr..

[19] New Zealand Qualifications Authority NCEA Factsheet. https://www.nzqa.govt.nz/assets/About-us/Publications/Brochures/NCEA-Factsheet-1-July-2017-FINAL.pdf.

[20] New Zealand Qualifications Authority NCEA Levels and Certificates. https://www.nzqa.govt.nz/ncea/understanding-ncea/how-ncea-works/ncea-levels-and-certificates/.

[21] Parnell W., Scragg R., Wilson N., Schaaf D., Fitzgerald E. (2003). NZ Food NZ Children: Key Results of the 2002 National Children’s Nutrition Survey.

[22] Statistics New Zealand Food Price Index: June 2023. https://www.stats.govt.nz/information-releases/food-price-index-june-2023/.

[23] Jones N.L., Gilman S.E., Cheng T.L., Drury S.S., Hill C.V., Geronimus A.T. (2019). Life Course Approaches to the Causes of Health Disparities. Am. J. Public Health.

[24] Hernandez J. In Fariness to Our Schools: Better Measures for Better Outcomes. https://www.nzinitiative.org.nz/reports-and-media/reports/in-fairness-to-our-schools/.

[25] Signal L.N., Walton M.D., Ni Mhurchu C., Maddison R., Bowers S.G., Carter K.N., Gorton D., Heta C., Lanumata T.S., McKerchar C.W. (2013). Tackling 'wicked' health promotion problems: A New Zealand case study. Health Promot. Int..

[26] New Zealand Qualifications Authority (2023). Annual Report NCEA, University Entrance and NZ Scholarship Data and Statistics 2022.

[27] Dearden L., Bouret S.G., Ozanne S.E. (2018). Sex and gender differences in developmental programming of metabolism. Mol. Metab..

[28] Aiken C.E., Ozanne S.E. (2013). Sex differences in developmental programming models. Reproduction.

[29] Savila F., Obolonkin V., Rush E. (2015). Tracking food consumption frequency of children from age 4 to 6 years: The Pacific Islands Families study. N. Z. Med. J..

[30] Giskes K., van Lenthe F., Avendano-Pabon M., Brug J. (2011). A systematic review of environmental factors and obesogenic dietary intakes among adults: Are we getting closer to understanding obesogenic environments?. Obes. Rev..

[31] Rush E. (2019). Wicked problems: The challenge of food safety versus food security-working towards the SDG goals?. Eur. J. Clin. Nutr..

